# One-Step Hydrothermal Synthesis, Thermochromic and Infrared Camouflage Properties of Vanadium Dioxide Nanorods

**DOI:** 10.3390/nano12193534

**Published:** 2022-10-10

**Authors:** Youbin Hao, Weidong Xu, Ming Li, Suhong Wang, Heng Liu, Xin Yang, Jie Yang

**Affiliations:** 1 Field Engineering College, Army Engineering University of PLA, Nanjing 210007, China; 2 Key Laboratory of Materials Physics, Anhui Key Laboratory of Nanomaterials and Nanotechnology, Institute of Solid State Physics, Chinese Academy of Sciences, Hefei 230031, China

**Keywords:** hydrothermal method, vanadium dioxide, thermochromic property, infrared camouflage

## Abstract

Vanadium dioxide (VO_2_) has attracted interest from researchers because it undergoes a metal–insulator phase transition (MIT), which is accompanied by a reversible and remarkable change in both electrical and optical properties. VO_2_ exhibits numerous polymorphs and thus it is essential to control the growth of specific monoclinic VO_2_ (M) and rutile VO_2_ (R) phases. In this study, we developed a cost-effective and facile method for preparing VO_2_ nanorods with a highly crystalline monoclinic phase by one-step hydrothermal synthesis, in which only V_2_O_5_ and H_2_C_2_O_4_ are used as raw materials. The phase evolution of VO_2_ during the hydrothermal process was studied. The obtained VO_2_ nanorods were thoroughly mixed with fluorocarbon resin and homogeneous emulsifier in an ethanol solution to obtain a VO_2_ dispersion. To prepare VO_2_ films, screen printing was performed with a stainless steel screen mesh mask on glasses or fabric substrate. The VO_2_ coating had good thermochromic performance; the infrared transmittance change was greater than 20% @1.5 μm whilst keeping the visible transmittance greater than 50%. Meanwhile, the polyester base coating on the fabric had an emissivity change of up to 22%, which provides a solution for adaptive IR camouflage.

## 1. Introduction

VO_2_ is the most common thermal-induced metal–insulator transition (MIT) material, which is characterized by a subtle and reversible structural transformation from VO_2_ (M) to VO_2_ (R) at the critical temperature (τ_c_) of ~68 °C [1,2,3] (“M” and “R” are abbreviations for monoclinic and rutile, respectively). The MIT of VO_2_ displays a significant change in its infrared optical property, which means that VO_2_ (M) can be transparent to infrared solar irradiation, and that VO_2_ (R) has an adverse effect on infrared irradiation at a temperature higher than τ_c_. Using this property, it can be observed that VO_2_ possesses excellent potential for a wide range of applications, including optic switches [4,5,6,7], energy-efficient smart windows [7,8,9], and infrared camouflage purposes [10,11,12]. In addition to VO_2_ (M/R), VO_2_ can also adopt other kinds of polymorphs, including monoclinic VO_2_ (B), triclinic VO_2_ (T), tetragonal VO_2_ (A), and parametricities VO_2_ (P). Among them, VO_2_ (M/R) is the most practical material due to its τ_c_ value that is close to ambient temperature. Furthermore, similar to most transition metals, vanadium exists in a wide range of oxidation states, which result in the existence of several intermediate vanadium oxides during the synthesis of VO_2_. Therefore, the study of a facile and suitable method of preparing the specific VO_2_ (M/R) phase presents many opportunities for optimization, with the main final goal of controlling the desirable size, morphology, and crystallinity of VO_2_ to satisfy different specific demands for practical applications. For example, for the sake of good dispersion, the quantity of VO_2_ (M/R) powder on the nanometer level is important; as regards the infrared modulation, the better the crystallization, the better the regulation ability.

Extensive studies have been conducted to prepare VO_2_ thin films or nanopowders, such as the sol–gel method [13], vapor phase transport [14], pulsed-laser deposition [15] hydrothermal treatment [16], and magnetron-sputter [17]. By contrast, hydrothermal treatment has been considered the main method used to prepare VO_2_ because it functions at both a high temperature and pressure that are well-suited to control the morphology, crystallinity, and chemical stoichiometry of the final product [18]. Unfortunately, to date, hydrothermal reactions in solution have mostly yielded the metastable phases of VO_2_ (B) or VO_2_ (A) because the reaction time is not long enough or the reaction temperature is not high enough, as shown in Figure S1. Therefore, the one-step hydrothermal process directly synthesizing thermodynamically stable phases of VO_2_ (M/R) has attracted significant attention in the research. 

The growth of VO_2_ (R) was first reported by the hydrothermal reaction of the V_2_O_3_-V_2_O_5_-H_2_O system at temperatures higher than 350 °C [19]. VO_2_ (R) nanorods were described by Jin et al. [20] in a study for which H_2_SO_4_ was used as a very effective addition for the morphology control of VO_2_. Gao et al. developed a process that could prepare single-crystal VO_2_ (R) snowflake powders by the hydrothermal treatment of V_2_O_5_ and oxalic acid at 240 °C for 7 days [21]. Micro- and nanocrystals of VO_2_(M)were prepared via the hydrothermal method using N_2_H_4_ as a reducing agent [22]. Wang et al. [23] confirmed that a certain quantity of W dopants could promote a phase transformation from VO_2_(B) to VO_2_(M/R) during hydrothermal synthesis; however, excess content can lead to the generation of undesirable crystals and a lower purity of VO_2_. Zou et al. [24] prepared VO_2_ nanoparticles via the hydrothermal treatment of NH_4_VO_3_, using N_2_H_4_·H_2_O as a reducing agent at 340 °C for 6h. The prepared powder was VO_2_ (M), including nanoparticles with particle sizes between 20 nm and 100 nm, and nanorods that were approximately 200 nm long. A novel, nested, hydrothermal reactor was proposed by Jin et al. in their study, where they separated the oxidant of hydrogen peroxide from the precursor solution; a reaction was performed at 260 °C for 24 h, and VO_2_(M) nanoparticles with an average size ∼30 nm were prepared [25]. In general, for the one-step hydrothermal synthesis, toxic hydrazine was extensively used to obtain VO_2_(M/R); however, sometimes other steps are essential to prepare the hydrothermal precursor. Thus, there still remains an opportunity to develop a facile and environmentally friendly hydrothermal route for synthesizing VO_2_ (M/R). While many studies report on VO_2_ nanoparticles, there is less literature concerning the one-step hydrothermal synthesis of VO_2_ nanorods for the exploration of thermochromic and infrared camouflage properties.

The main purpose of the current study was to directly perform the hydrothermal synthesis of VO_2_(M/R), in which only V_2_O_5_ and H_2_C_2_O_4_ were used as raw materials. To prepare VO_2_ films, we performed screen printing with a stainless steel screen mesh mask on glasses or a fabric substrate. The VO_2_ coating had an obvious infrared modulation ability and displayed a good thermochromic performance. Moreover, the polyester base coating on fabric presented an emissivity change of up to 22%, which provides great potential for adaptive infrared camouflage applications.

## 2. Materials and Methods

### 2.1. Materials and Synthesis

In the experiments, all of the chemicals, including V_2_O_5_ (99.7%, Sarn Chemical Technology, Shanghai, China), C_2_H_2_O_4_·2H_2_O (analytical purity, Shanghai Qianshun Chemical Reagent, Shanghai, China), C_2_H_5_OH (99.7%, Shanghai Titan, Shanghai, China), and Disperbyk-110 (copolymer-containing acid group, 52% nonvolatile component, BYK, Shanghai, China) were used directly, without further purification. In a typical hydrothermal process, 1.82 g of V_2_O_5_ powder was added to 60 mL of deionized water at room temperature and stirred thoroughly for 2 h. The solution changed from brownish yellow to a blue-green clarified solution. Then, different quantities of C_2_H_2_O_4_·2H_2_O were added to the solution and continuously stirred until the C_2_H_2_O_4_·2H_2_O was completely dissolved. According to the different molar ratios between C_2_H_2_O_4_·2H_2_O and V_2_O_5_ of 0.5:1,1:1,1.5:1,2:1,2.5:1 and 3:1, the samples were labeled as S1, S2, S3, S4, S5, and S6, respectively. The obtained precursor solution was transferred into a 100 mL PPL material-lined stainless-steel autoclave, and the reactor was sealed. The hydrothermal temperature was set to 280 °C and the reaction time was 48 h. The samples were cooled to room temperature in air at the end of the reaction process. A black precipitate was collected by centrifugation and washed three times with deionized water and ethanol in turn. The final black powder solution was obtained after drying in a vacuum oven at 60 °C for 12 h. There was no subsequent annealing treatment.

### 2.2. Preparation of VO_2_ Coatings

#### 2.2.1. Preparation of VO_2_ Coating on Polyester Fabric Surface

A total of 1 g of VO_2_ powder and 0.1 mL of Disperbyk-110 dispersant were mixed together in 10 mL of ethanol and sonicated for 30 min, and then a high shear emulsifier was added to completely disperse the VO_2_ powder. A total of 2 g of resin (fluorocarbon resin, PVP, aqueous polyurethane) was used to obtain the ink for printing. The screen-printing technique was used to print the VO_2_ coating evenly on the polyester fabric. The screen used for the printing process was 200 mesh to ensure that the VO_2_ nanopowders could be smoothly printed on the surface of the base fabric. The printed fabric was dried on a constant-temperature heating table at 50 °C for 20 min to remove the solvent to form a uniform and firm coating.

#### 2.2.2. Preparation of VO_2_ Coating on Quartz Surface

A spin coater was used to prepare VO_2_ optical coatings on the surface of quartz glass using the same ink as mentioned above. The spin-coater speed was set at 400 r/min, acceleration at 100 r/s, and spin-coating time at 65 s. The coatings were prepared with different thicknesses from one to four spin coatings.

### 2.3. Characterization 

An X-ray diffractometer (RIKEN SmartSab 9kw, Wakō, Japan) was used to characterize the phase composition and crystalline structure of the samples, with a CuKα source, a target voltage of 40 kv, a target current of 30 mA, a scan range of 5°~90°, a scan speed of 5 (°)/min, and a scan length of 0.2°. The surface morphology of the samples was characterized by field-emission electron microscopy (Zeiss MERLIN and FEI Quanta 400 Feg, Jena, Germany). A differential scanning calorimeter (NETZSCH DSC 214, Selb, Germany) was used to characterize the phase-transition behavior of the samples upon a heating and cooling rate of 10 °C/min under an N_2_ atmosphere. An IR-2 infrared emissometer (Shanghai Institute of Technical Physics, Shanghai, China) was applied to analyze the normal integrated emissivity of fabrics in the 8–14 μm wavelength range. All the emissivity values were obtained as an average of three measurements. The infrared thermogram of the coating was obtained using an infrared thermographic camera (FLIR E5xt, Wilsonville, OR, USA).

## 3. Results and Discussion

### 3.1. VO_2_ Nanorods

**Figure 1:**
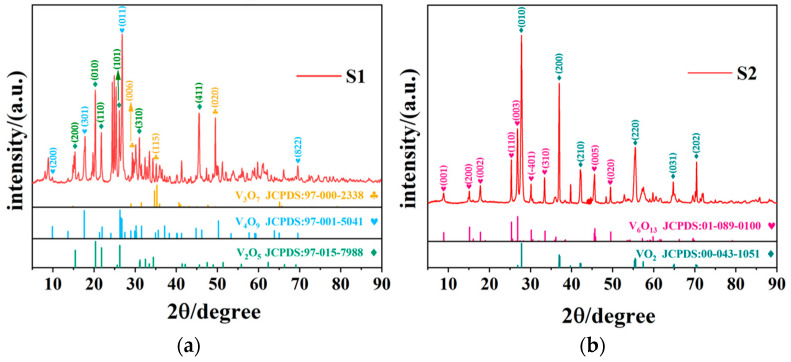
XRD data of (**a**) S1 (reactants ratio 0.5:1) and (**b**) S2 (reactants ratio 1:1) samples.

Figure 1 presents the XRD plots of S1 and S2 samples; evidently, the insufficient quantity of oxalic acid cannot reduce all V_2_O_5_ to VO_2_ at a ratio lower than 1:1. For S1, it was a mixture phase of V_2_O_5_ (JCPDS Card No. 97-015-7988), V_4_O_9_ (JCPDS Card No. 97-001-5041), and V_3_O_7_ (JCPDS Card No. 97-000-2338). The diffraction peaks at 2θ of 15.424°, 20.352°, 21.787°, 26.221°, 31.070°, and 45.605° correspond to (200), (010), (110), (101), (310), and (411) of V_2_O_5_, respectively; peaks at 2θ of 9.860°, 17.575°, 26.292°, 37.143°, 50.211°, and 69.522° belong to (200), (301), (011), (412), (020), and (822) of V_4_O_9_, respectively; and the (200), (600), (-111), (006), and (020) crystal planes of V_3_O_7_ present diffraction peaks at 8.099°, 24.462°, 24.926°, 29.334°, and 49.512°, respectively. For S2, all of the three phases mentioned above disappeared, and vanadium oxide was reduced further to lower valence states of V_6_O_13_ (JCPDS Card No. 01-089-0100) and VO_2_ (JCPDS Card No. 00-043-1051). The characteristic diffraction peak at 27.796° corresponded to the (011) crystalline plane of VO_2_ (M), while the peaks at 2θ = 8.873°, 15.125°, 17.800°, 25.359°, and 26.838° can be assigned to the (001), (200), (002), (110), and (003) crystal faces of V_6_O_13_, respectively. The crystallographic data of the vanadium oxides observed in S1 and S2 are presented in Table 1.

Generally, the S1 sample indicates that the low concentration of oxalic acid is not sufficient to completely reduce V_2_O_5_, and some unreacted V_2_O_5_, as well as intermediate products of V_4_O_9_ and V_3_O_7_, coexist in S1, while VO_2_ (M) is evident in S2, but the intermediate product of V_6_O_13_ remains present. Therefore, a higher concentration of oxalic acid is necessary to obtain pure VO_2_.

**Figure 2:**
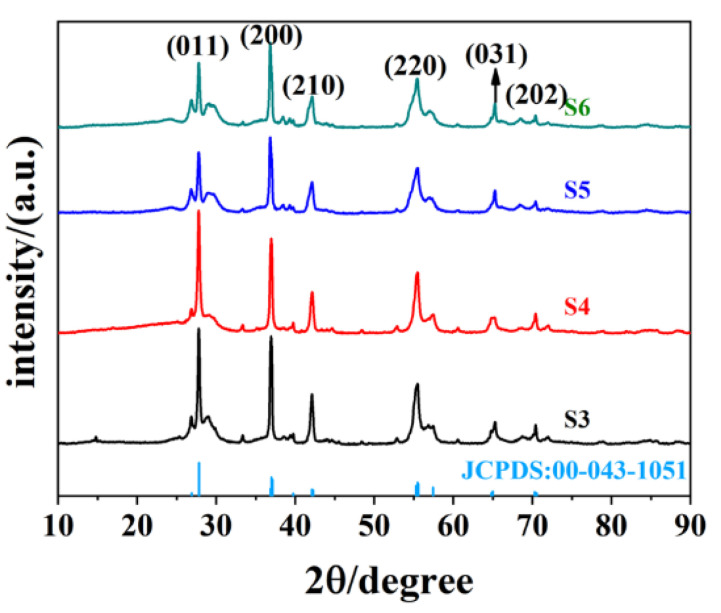
XRD data for the hydrothermal product with different molar ratios between C_2_H_2_O_4_·2H_2_O and V_2_O_5_, and ratios for S3–S6 are 1.5:1, 2:1, 2.5:1, and 3:1, respectively.

**Figure 3:**
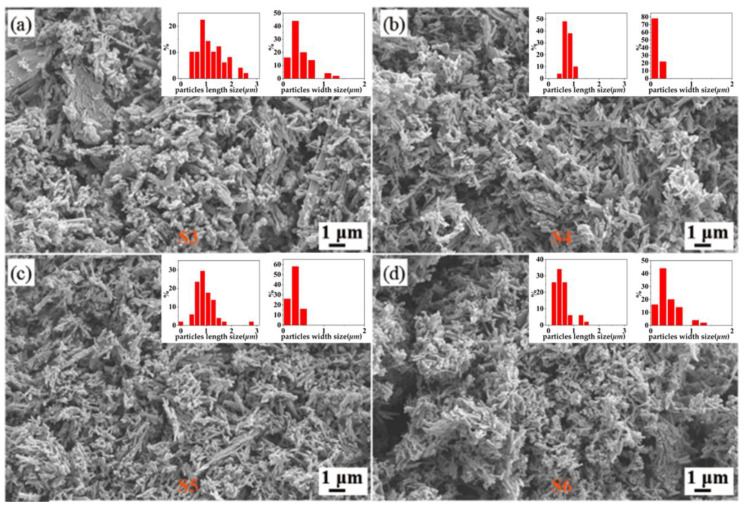
SEM images of (**a**) S3 (reactants ratio 1.5:1), (**b**) S4 (reactants ratio 2:1), (**c**) S5 (reactants ratio 2.5:1), and (**d**) S6 (reactants ratio 3:1) samples.

Figure 2 presents the XRD data for the hydrothermal product when the precursor ratio between C_2_H_2_O_4_·2H_2_O and V_2_O_5_ is greater than 1:1; all of the products are VO_2_ (M). The diffraction peaks at 2θ = 27.796°, 37.089°, 42.079°, 55.451°, 64.972°, and 70.256° for the four samples corresponded to the (011), (200), (210), (220), (031), and (202) crystalline planes of VO_2_ (M), respectively, which indicates the purity of the obtained samples. By contrast, the diffraction peak of the S4 sample was higher than that of the other three of samples, implying the good crystalline properties of S4. Meanwhile, as can be observed from S5 and S6, the much higher concentration of oxalic acid is not beneficial to the purity of VO_2_ and leads to the poor crystallinity of the samples. Overall, the main XRD peaks of these four samples are basically consistent. This kind of consistency is also reflected in their morphology, as presented in the SEM in Figure 3. All of four samples (S2–S5) presented short, rod-like structures, but some differences still remained. For S3, there were obviously some larger micron-scale layers and lumps of crystals, and the particle size was not uniform across the sample. A small amount of flakes close to the micron scale were also observed in S5 and S6, suggesting that excessive oxalic acid was not beneficial to forming uniform samples. The nanorods present in S4 were much more uniform and presented good dispersity properties.

**Figure 4:**
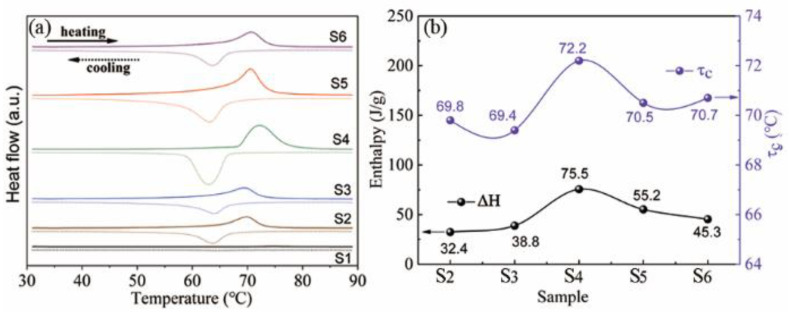
(**a**) DSC data of samples with different oxalic acid concentrations; (**b**) phase-change enthalpy (ΔH) and transition temperature ($\tau_{c}$) versus hydrothermal duration.

Figure 4 presents the DSC data obtained for samples of S1–S6. Distinct endothermic and exothermic peaks were observed during the heating and cooling cycles, respectively, for samples S2–S6. The thermal hysteresis indicates fully reversible and first-order phase-transition features. The phase-transition temperatures and enthalpies of the samples were determined by particular software (NETZSCH Proteus Thermal Analysis). As shown in Figure 4b, τ_c_ is mainly concentrated at approximately ~70 °C. It can be observed that sample S4 presents the highest τ_c_ of 72.2 °C, indicating the good crystallinity, which is consistent with the XRD data. The underlying mechanism was mainly related to the defects that can act as the nucleation site to trigger the phase transition from VO_2_(M) to VO_2_(R) [26]. S4 presents relatively fewer defects and thus produces a higher τ_c_ value. The highest phase-change enthalpy (ΔH) for S4 also indicates that it presents the best crystallinity (Figure 4b). The higher enthalpy presented during the phase transition indicates that the infrared modulation property of sample S4 will considerably change, which is reflected by the fact that its emissivity changes more rapidly than the other samples. Therefore, in the subsequent experiment, sample S4 was selected for the preparation of a VO_2_ coating.

### 3.2. Infrared Camouflage and Thermochromic Properties

VO_2_ coating is printed on the surface of polyester fabric using screen-printing technology. Polyester fabric is, at present, the most widely used fabric material for the factory production of camouflage nets because it is a high-strength fiber with good strength and toughness properties that is not easily damaged, and it is heat resistant and not easily deformed. Screen-printing technology is a convenient and practical printing technology that can use various types of inks, such as oil-based, water-based, synthetic-resin emulsion type, and powder; at the same time, it is not limited by the shape of the substrate’s surface and the size of the area, which exhibits a high operability quality and is suitable for a wide range of purposes and uses.

**Figure 5:**
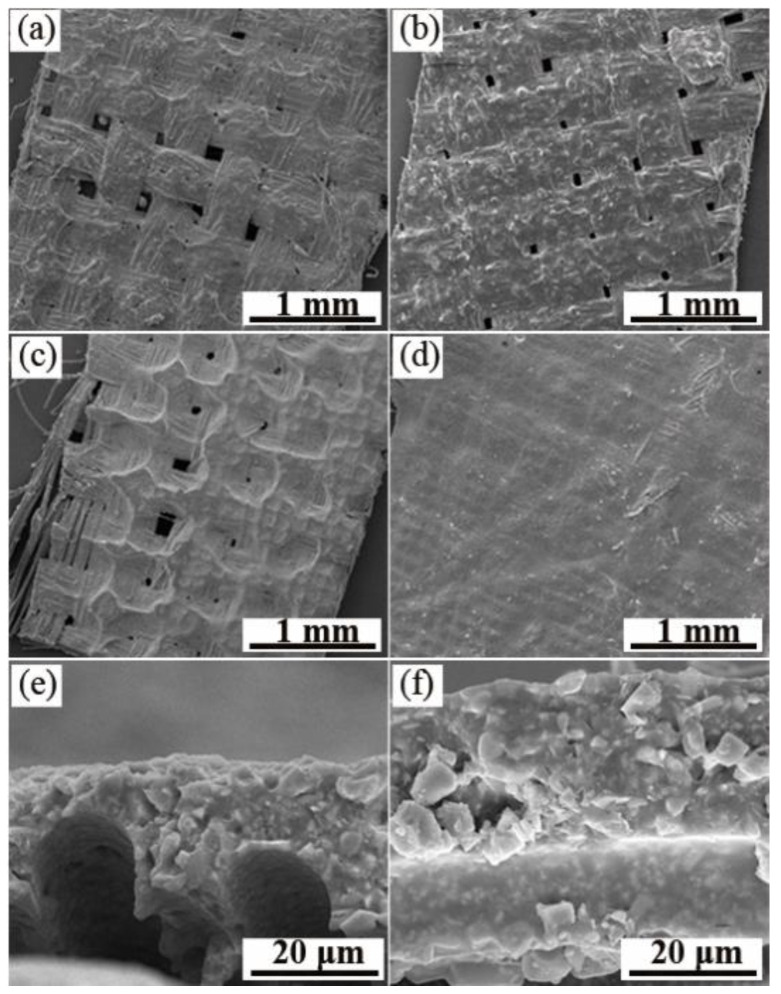
SEM images of the surface and interface of the coating. Coating surface on which the film-forming substance is present; (**a**) waterborne polyurethane; (**b**) PVP; (**c**) fluorocarbon resin; (**d**) fluorocarbon-resin-coated surface printed twice; (**e**) fluorocarbon-resin-coated cross-section printed once; and (**f**) fluorocarbon-resin-coated cross-section printed twice.

In the current paper, three kinds of resins commonly used to prepare infrared stealth coatings were used to prepare the coatings: aqueous polyurethane (PU) [27], PVP [28], and fluorocarbon resin [29]. The advantage of the three resins is that they are all infrared transparent resins. The resins do not affect the infrared properties of the prepared coatings, and the sample powders can be well dispersed in the three resins. However, PU and PVP are waterborne resins, and the coating stability following film formation is not as good as that of fat-soluble fluorocarbon resin. The SEM images of the printed coating are presented in Figure 5a–c. It can be observed from the figure that the coating surface of water-based polyurethane is rough and cannot completely cover the fibers of the polyester base cloth; the coating surface of the PVP resin is smooth but there are a lot of bubbles present. The coating surface of the fluorocarbon resin is more delicate, and the VO_2_ powder is evenly adhered to the fiber surface. Therefore, the fluorocarbon resin was finally elected as the film-forming substance for the coating preparation. Figure 5c,d present the SEM images of the surface and cross-section of the coating formed by printing once and twice, respectively. The surface fibers of the twice-printed coating are completely covered by the coating, and the surface is fine without cracks and bubbles. From the cross-sectional view, it can be observed that the average thickness of the coating is approximately 10 μm for the once-printed coating and approximately 20 μm for the twice-printed coating, doubling the thickness.

**Figure 6:**
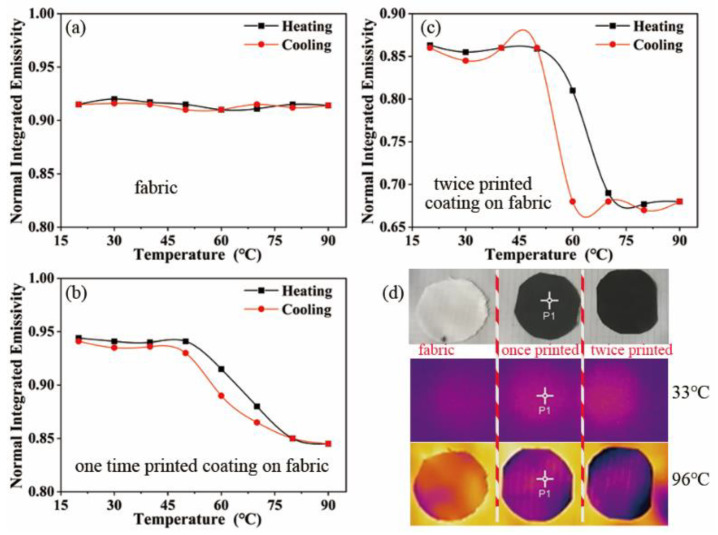
(**a**) Emissivity of polyester base fabric at different temperatures; (**b**) emissivity of once-printed coatings at different temperatures; (**c**) emissivity of twice-printed coatings at different temperatures; (**d**) from top to bottom, the image presents the optical image, and normal-temperature and high-temperature thermal infrared images.

Figure 6a–c presents the values of the surface’s normal, integrated emissivity of the polyester base fabric, and the once- and twice-coated films using an IR-2 dual-band emissivity tester. The emissivity-modulation ability of the coating increased significantly with the increase in the coating’s thickness. The maximum change in the emissivity of the once-printed coating was 0.1, reaching 10.6%; the maximum change in the emissivity of the twice-printed coating was 0.19, reaching 22%; and the surface of the twice-printed coating was smoother and flatter; therefore, its room-temperature emissivity was lower than that of the polyester-based fabric.

The thermal infrared camouflage capability of the coating was characterized using infrared thermography. Figure 6d presents the optical photographs of the polyester base fabric, once-printed coating, as well as the twice-printed coating, with three samples placed on the same constant-temperature heating table. The figure presents the infrared thermal image at room temperature, and the uncoated sample has almost the same thermal radiation intensity as the coated sample. When the temperature reaches 96 °C, the coated sample has a significantly lower thermal radiation intensity and can blend better with the background, achieving the effect of adaptive camouflage.

**Figure 7:**
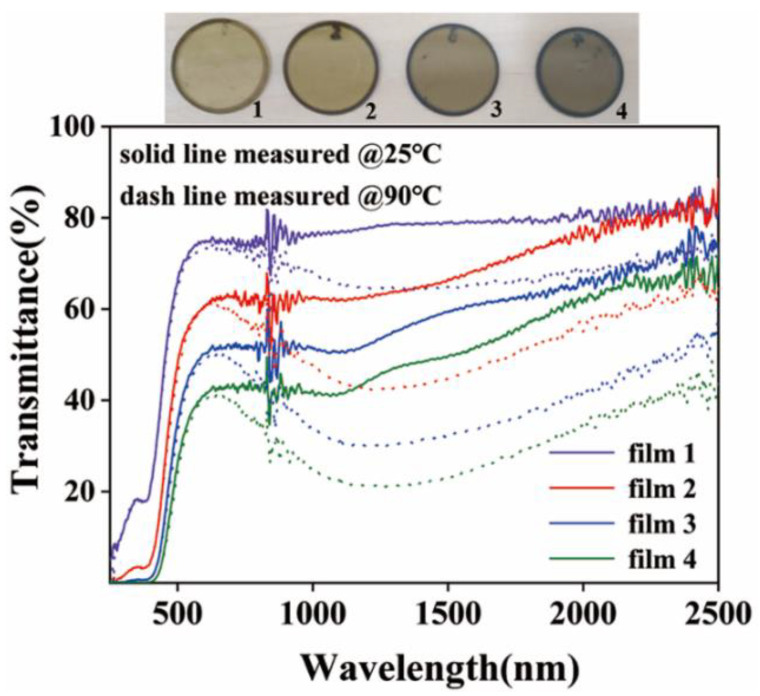
Images and optical transmittance of samples with different spin-coating times.

Figure 7 exhibits the optical transmittance curves of the coatings with different thicknesses formed by spin coating on the surface of quartz glass using the above-selected inks, in which the solid and dashed lines indicate the low- and high-temperature cases, respectively. The four samples were spin-coated 1–4 times, numbered 1–4 and in order, and the transmittance curves present that the low-temperature transmittance decreases with the increase in the coating’s thickness, from approximately 80% to less than 60%, but the optical-transmittance-modulation ability of the coating continuously increases with the increase in the thickness. The amount of infrared transmittance changes for samples 1, 2, 3, and 4 were 14%, 22%, 27%, and 29% @1.5 μm, respectively, while maintaining the visible transmittance at a value greater than 50%. Ru Chen et al. also prepared VO_2_ films in their study. The infrared transmittance changes in the films printed once and twice were 20% and 23%, respectively [30]. The thermochromic properties of the films prepared in our study are comparable to those presented in the literature.

## 4. Conclusions

The VO_2_ nanorods were synthesized by a one-step hydrothermal method using only V_2_O_5_ and H_2_C_2_O_4_ as the raw materials, and the optimum mole ratio between C_2_H_2_O_4_ and V_2_O_5_ was 2:1. Three different polymers were used to prepare the VO_2_ ink that was used to print the coating onto the surface of the polyester base cloth, and fluorocarbon resin was identified as the best dispersant. The flexible VO_2_ coating can adaptively change the surface’s emissivity property, and the changes in emissivity could reach 0.19, which presented good adaptability to the environment and was expected to be used for producing military tents, camouflage nets, and other equipment. The VO_2_ coating on the glass substrate also displayed a good thermochromic performance; the amount of infrared transmittance change was greater than 20% @1.5 μm while maintaining the visible transmittance value at a figure greater than 50%.

## Figures and Tables

**Table 1:** Crystallographic data of different products in the sample.

Obtained Sample	Space Group	Cell Parameters a, b, c (Å)
V_4_O_9_	Pnma(62)	17.926, 3.631, 9.396
V_3_O_7_	C2/c(15)	21.921, 3.679, 18.341
V_6_O_13_	C2/m	11.960, 3.713, 10.070
VO_2_ (M)	P2_1_/c	5.752, 4.538, 5.383

## Supplementary Materials

The following supporting information can be downloaded at: https://www.mdpi.com/article/10.3390/nano12193534/s1, Figure S1: Hydrothermal product of VO_2_ versus hydrothermal temperature and reaction time; Table S1: the crystallography information of VO_2_ polymorphs.
