# Supplementary material for: One-Step Hydrothermal Synthesis, Thermochromic and Infrared Camouflage Properties of Vanadium Dioxide Nanorods
Source: Nanomaterials (Basel). 2022 Oct 10;12(19):3534. doi: 10.3390/nano12193534 (PMC9565468; doi:10.3390/nano12193534)
Supplement: Supplementary file 1 [file nanomaterials-12-03534-s001.zip › nanomaterials-1933870-supplementary.pdf]

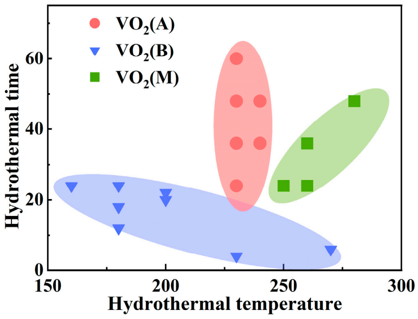

Figure S1 . Hydrothermal product of VO_2 versus hydrothermal temperature and reaction time

Table S1. the crystallography information of VO2 polymorphs.

Polymorphs	Unit cell parameters a,b,c (Å)	β(°)	Space group
VO_2(B)	12.054,3.693,6.424	106.96	C2/m
VO_2(A)	8.450,8.450,7.678	90	$P4_2$/ncm
VO_2(M)	5.752,4.538,5.383	122.64	$P2_1$/c
VO_2(R)	4.554,4.554,2.856	90	P42/mnm
VO_2(D)	4.597,5.684,4.913	89.39	P2/c
VO_2(P)	4.890,9.390,2.930	90	Pbnm
